# F201. KINASE NETWORK DYSREGULATION IN SCHIZOPHRENIA: IMPLICATIONS FOR NEW TREATMENT STRATEGIES

**DOI:** 10.1093/schbul/sby017.732

**Published:** 2018-04-01

**Authors:** Eduard Bentea, Erica Depasquale, Jarek Meller, Zhexing Wen, Robert E McCullumsmith

**Affiliations:** 1 University of Cincinnati; 2 Cincinnati Children’s Hospital Medical Center; 3 Emory University School of Medicine

## Abstract

**Background:**

Disrupted-in-schizophrenia 1 (DISC1) is one of the most substantiated genetic risk factors for schizophrenia (SZ). A large array of animal studies supports an etiopathogenic role of DISC1, by linking it with regulation of processes such as synapse formation and neuronal development. However, much less is known regarding the involvement of DISC1 in human neurons. Induced pluripotent stem cells (iPSCs) generated from patients carrying the disease have emerged as powerful tools to study cellular dysfunction in a disease-relevant context. In this study, we investigated serine/threonine kinase networks in a human iPSC model of DISC1-related SZ.

**Methods:**

PamChip arrays evaluate kinase activity by measuring phosphorylation levels of a series of immobilized peptide sequences during exposure to kinases in the sample. We employed PamChip arrays to map the serine/threonine sub-kinome of neuronally differentiated iPSCs generated from a patient with SZ presenting the frame-shift DISC1 mutation (D2-1), an unaffected family member without the mutation (C3-1), as well as of isogenic iPSC lines in which the mutation was either corrected in D2-1 (resulting in the cell line D2-R), or introduced in C3-1 (resulting in the cell line C3-M). Using a bioinformatics workflow that identifies kinase hits using a random sampling model, we identified kinases that emerged as common hits after comparing D2-1 with D2-R (changed after rescuing the mutation in the patient cell line) and C3-M with C3-1 (changed after introducing the mutation in the control cell line). We used the resulting kinase network to identify pathways, perturbagens, and drugs related to the disease phenotype.

**Results:**

By comparing D2-1 to D2-R, 9 peptide sequences were identified to be differentially phosphorylated at a +/- 1.15 fold-change level. After assigning upstream kinases to these peptides and generating the random sampling model, we identified 3 kinase subfamilies which were over-represented in D2-1 vs. D2-R: TAO, KHS and 5’ adenosine monophosphate-activated protein kinase (AMPK). By comparing C3-M to C3-1, we could identify 13 peptide sequences differentially phosphorylated at a +/- 1.15 fold-change level. Mapping these sequences to upstream kinases and running the random sampling model, led to the identification of 9 kinase subfamilies over-represented in C3-M vs. C3-1: AMPK, TAO, BUD32, WNK, KHS, RAD53, CK1, NEK and MLK. By overlapping the results, we could identify a set of 3 kinase subfamilies (TAO, KHS, and AMPK) commonly changed between the two methods of comparison. Ingenuity pathway analysis identified post-translational modification, cell signaling, cell morphology, cell cycle, and cellular assembly and organization, as the top functions of the DISC1 kinase network.

**Discussion:**

Kinases are potent modulators of intracellular signaling that control patterns of gene expression, cytoskeletal dynamics, function of neurotransmitter systems and cellular metabolism, which may be of relevance to the etiopathogenesis of mental disorders, such as SZ. Herein, we characterized the serine/threonine sub-kinome of neuronally differentiated iPSCs from a patient with SZ presenting with a 4-bp deletion in DISC1. Using gene editing we created isogenic cell lines to either rescue the mutation in the patient cell line, or introduce the mutation in iPSCs obtained from an unaffected family member, to strengthen causality for the DISC1 mutation. This approach led to the identification of 3 kinase subfamilies as common hits of the DISC1 phenotype: TAO, KHS, and AMPK. Our unbiased approach led to the novel identification of kinases implicated in DISC1-related SZ. Further validation of these findings may open new avenues for treating this highly disabling neuropsychiatric disorder.

